# Bushfire smoke: urgent need for a national health protection strategy

**DOI:** 10.5694/mja2.50511

**Published:** 2020-02-23

**Authors:** Sotiris Vardoulakis, Bin B Jalaludin, Geoffrey G Morgan, Ivan C Hanigan, Fay H Johnston

**Affiliations:** ^1^ National Centre for Epidemiology and Population Health Research School of Population Health Australian National University Canberra ACT; ^2^ Ingham Institute for Applied Medical Research University of New South Wales; ^3^ School of Public Health and University Centre for Rural Health University of Sydney Sydney NSW; ^4^ Health Research Institute University of Canberra ACT; ^5^ Menzies Institute for Medical Research University of Tasmania Hobart TAS

**Keywords:** Air pollutants, Climate change, Population health

More nuanced health advice is needed to protect populations and individuals from exposure to bushfire smoke

Bushfires have always been a feature of the natural environment in Australia, but the risk has increased over time as fire seasons start earlier, finish later, and extreme fire weather (ie, very hot, dry and windy conditions that make fires fast moving and very difficult to control) becomes more severe with climate change.^1^, ^2^, ^3^ The 2019–20 bushfires in Australia, particularly in New South Wales, Victoria, Queensland and the Australian Capital Territory, have caused at least 33 fatalities, extensive damage to property and destruction of flora and fauna, and have exposed millions of people to extreme levels of air pollution. Bushfire smoke, as well as smoke from prescribed burns, contains a complex mixture of particles and gases that are chemically transformed in the atmosphere and transported by the wind over long distances.^4^ In this context, a major public health concern is population exposure to atmospheric particulate matter (PM) with a diameter < 2.5 μm (PM_2.5_), which can penetrate deep into the respiratory system, inducing oxidative stress and inflammation,^5^ and even translocate into the bloodstream.^6^

Such exposure can adversely affect health outcomes. Mortality rates have been found to increase in Sydney on days with high bushfire smoke pollution.^7^ Hospital admissions, emergency department attendances, ambulance call‐outs and general practitioner consultations, particularly for respiratory conditions, all increase during periods of severe PM_2.5_ levels from bushfires.^8^, ^9^, ^10^, ^11^ The risks from air pollution are amplified when combined with high temperatures during heatwaves, with an increased effect on mortality.^12^

Certain population groups are at higher risk from exposure to smoke, either because they typically breathe in more air per bodyweight and their organs are still developing (young children), spend more time outdoors (outdoor workers, homeless people), or are more vulnerable to smoke due to old age or a pre‐existing health condition (asthma, chronic obstructive pulmonary disease or other respiratory condition, cardiovascular illness, or diabetes). There is evidence that exposure to bushfire smoke during pregnancy is associated with reduced birthweight in babies and a higher risk of gestational diabetes in mothers.^13^, ^14^ People in lower socio‐economic groups are potentially at higher risk, as they may have poorer housing, and lower health literacy and ability to take preventive measures.

## Health protection advice and trade‐offs

Current health protection advice related to bushfire smoke mainly focuses on short term measures aimed at reducing personal exposure to pollution. This includes advice to stay indoors with windows and doors closed, and reduce strenuous physical exercise outdoors, particularly if individuals experience health symptoms or have pre‐existing respiratory or cardiovascular conditions, when PM_2.5_ concentrations are increased. The PM_2.5_ national standard of 25 μg/m^3^ measured as a 24‐hour mean (National Environment Protection (Ambient Air Quality) Measure: https://www.legislation.gov.au/Details/F2016C00215) is consistent with the World Health Organization's air quality guidelines.^15^ However, PM_2.5_ concentrations presented as hourly averages are more useful for planning daily activities, as these better reflect current air quality, which can change rapidly during bushfire episodes. Currently, state and territory government departments use a range of different air quality metrics (such as a composite Air Quality Index based on multiple pollutants), averaging times and thresholds to stratify health messages into colour‐coded bands (very good, good, fair, poor, very poor, hazardous). The discrepancies in the presentation of this air quality information and related health advice across jurisdictions is confusing for the public.

General advice also includes having access to regular medication, such as asthma medication, checking on older neighbours, and seeking medical attention if needed. Such advice, however, has been tailored to brief air pollution episodes that last only a few hours or days. In situations like the 2019–20 bushfire smoke events in eastern Australia, where severe smoke pollution persists over longer periods (weeks to months) and affects large population centres, there is a need for more nuanced and detailed health advice based on location‐specific air quality data and forecasts.

Reducing prolonged or heavy physical exercise outdoors may become impractical over longer periods; for example, for school children and outdoor workers. Children and adults need to carry out a range of daily activities that involve spending time outdoors. Advice to reduce strenuous physical exercise outdoors becomes problematic over longer periods, owing to the recognised health benefits from active travel (ie, walking and cycling) and regular outdoor exercise, and potential lack of access to indoor sports facilities. We believe that more nuanced advice would encourage individuals to be guided by location‐specific air quality forecasts and the pattern of hourly PM_2.5_ concentrations at nearby air quality monitoring locations, and to plan their daily activities in ways that minimise exposure to pollution.

For example, PM_2.5_ levels were lower in most locations in Sydney in early morning hours during the December 2019 bushfire smoke episode (Box 1, A). Exercising outdoors and cycling or walking to school or work within this time window would help maintain good physical activity levels without substantially increasing exposure to smoke. Locations in the city's north were affected by much higher PM_2.5_ concentrations than some locations in the south at the highest peak of smoke on 10 December 2019 (Box 1, B). Real time information on the temporal and spatial variation of air pollution in all jurisdictions should be made available online and through other media to enable individuals to assess nearby air quality. Avoiding pollution from other sources (road traffic, cigarette smoking, etc) is also advisable, although widespread bushfire smoke is likely to dominate personal exposure to PM_2.5_ during severe smoke events.

Box 1 **Hourly average PM_2.5_ levels, Sydney region, December 2019**

PM_2.5_ = atmospheric particulate matter with a diameter < 2.5 μm. **A**: Hourly average PM_2.5_ data between 1 and 22 December 2019, downloaded for 15 air quality monitoring stations in the Sydney region from the New South Wales Department of Planning, Industry and Environment database (https://www.dpie.nsw.gov.au/air-quality/search-for-and-download-air-quality-data). The straight line represents the Australian PM_2.5_ standard of 25 μg/m^3^ measured as a 24‐hour mean (National Environment Protection (Ambient Air Quality) Measure). Note that full data validation has not been completed for these records and they have only passed an initial automated validation process. **B**: Hourly average PM_2.5_ levels (μg/m^3^) at monitoring stations at the peak of the bushfire smoke event on 10 December 2019 at 1 pm.

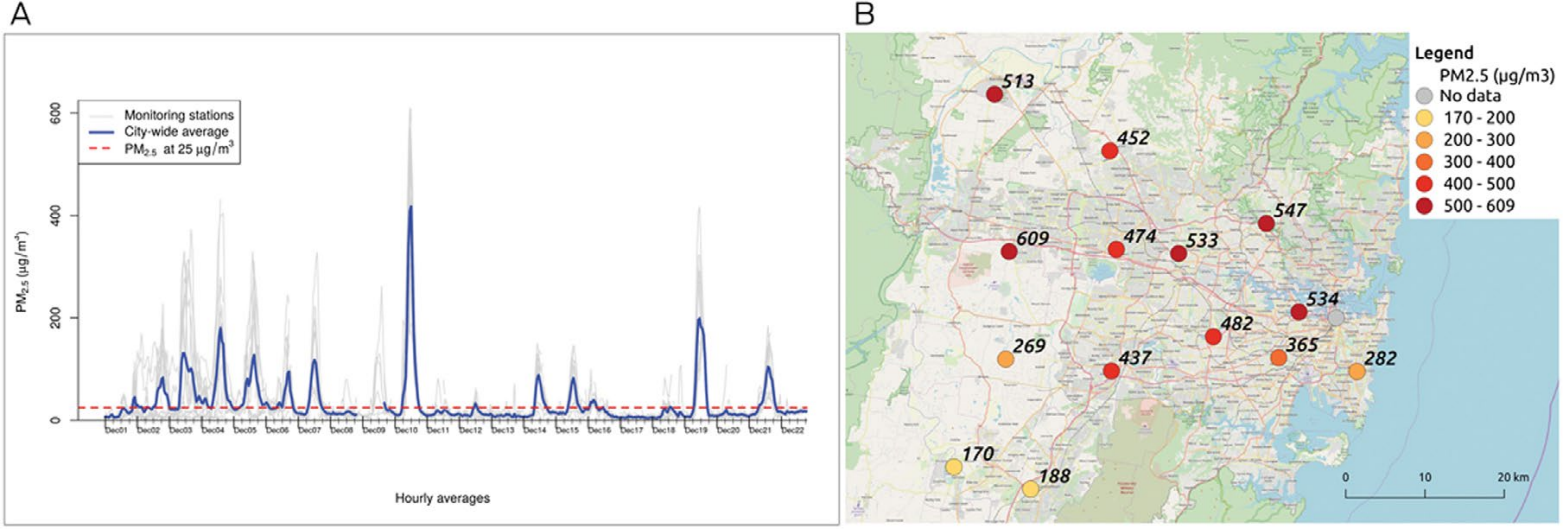

Advice to stay indoors may be ineffective over longer periods. Older houses in Australia are often quite “leaky”, allowing bushfire smoke to penetrate indoors over time and creating unhealthy indoor air quality conditions. Well sealed and air conditioned indoor environments (typically, modern apartments and offices, libraries and shopping centres) can provide respite from smoke pollution, particularly if effective air filtration systems are in place. However, many urban residents exposed to bushfire smoke, such as older people and those with restricted mobility, may not have easy access to such places.

Temporary relocation to a different area or city could reduce exposure to air pollution during localised but persistent smoke episodes. However, relocation has its own risks and is impractical (particularly for older people and for those with cognitive or mobility problems), especially when larger geographical areas are affected by smoke.^16^ Temporary relocation to a dwelling with better indoor air quality (eg, a modern air conditioned apartment) within the same neighbourhood may be a more practical and less stressful solution for those at higher risk (eg, people with severe asthma, pregnant women, and older people).

The priority for those affected should be to create a clean air space within their home, by sealing doors and windows and using air conditioning and filtration if possible, where they can spend most of their time during prolonged periods of bushfire smoke.^17^ However, many people may not be able to afford air conditioning and filtration units. Homes should be ventilated during periods of cleaner outdoor air quality (eg, around midnight in Sydney in December 2019; Box 1, A), to cool down the homes and avoid build‐up of indoor pollutants.

Access to regular medication, including asthma preventers and relievers, statins or aspirin, is important for people with pre‐existing lung and heart conditions, and should be arranged in consultation with their GPs. Maintaining a healthy diet, with plenty of fruit and vegetables, and keeping well hydrated is likely to help reduce short and long term health effects. There is suggestive evidence that antioxidant and fish oil supplementation and dietary intake may have a protective effect against air pollution exposure;^18^, ^19^ however, more research is needed to support this.

Much of the media attention during periods of bushfire smoke relates to the use of facemasks. These are increasingly used by the general public in highly polluted Asian cities, particularly in China.^20^ Use of facemasks during brief air pollution episodes (outside occupational settings and extreme air pollution emergencies related to volcanic eruptions) is not routinely recommended by health authorities. This is because their effectiveness depends heavily on the facial fit, material and condition of the masks. Surgical masks may have reasonable filtration efficiency; however, their design generally confers poor facial fit and high inward leakage of PM_2.5._ 20 Professional P2 or N95 facemasks, which can provide very efficient filtration of PM_2.5_ if well fitted, are only designed for adults and can make breathing more difficult and increase thermal discomfort.^20^ More research is needed on the longer term health benefits and potential drawbacks of different types of facemasks for adults and children. Such masks do not confer protection from exposure to toxic gases in bushfire smoke (eg, carbon monoxide, nitrogen oxides and volatile organic compounds) that may be present closer to the fire front. There are a number of practical, medical and ethical considerations that should ultimately inform a decision about whether or not to recommend and distribute facemasks to the general public, outdoor workers and sensitive groups during air pollution emergencies.^21^, ^22^ Clear information about the effectiveness, benefits and drawbacks of different types of masks should be provided by health authorities to enable individuals, health professionals and employers to make informed decisions.

## Risk communication

Nuanced and balanced public health communication that takes into account health risks, people's concerns and the effectiveness and practicality of protective measures is needed. Bushfire smoke alerts, real time air quality data and forecasts, and related health protection advice (Box 2) can help to reduce population exposure to hazardous air pollution, by enabling individuals, particularly those more sensitive, to plan their daily activities accordingly.

Box 2 **Factsheet: bushfire smoke and health protection**

Source: Australian National University Research School of Population Health (https://rsph.anu.edu.au/news-events/news/how-protect-yourself-and-others-bushfire-smoke).

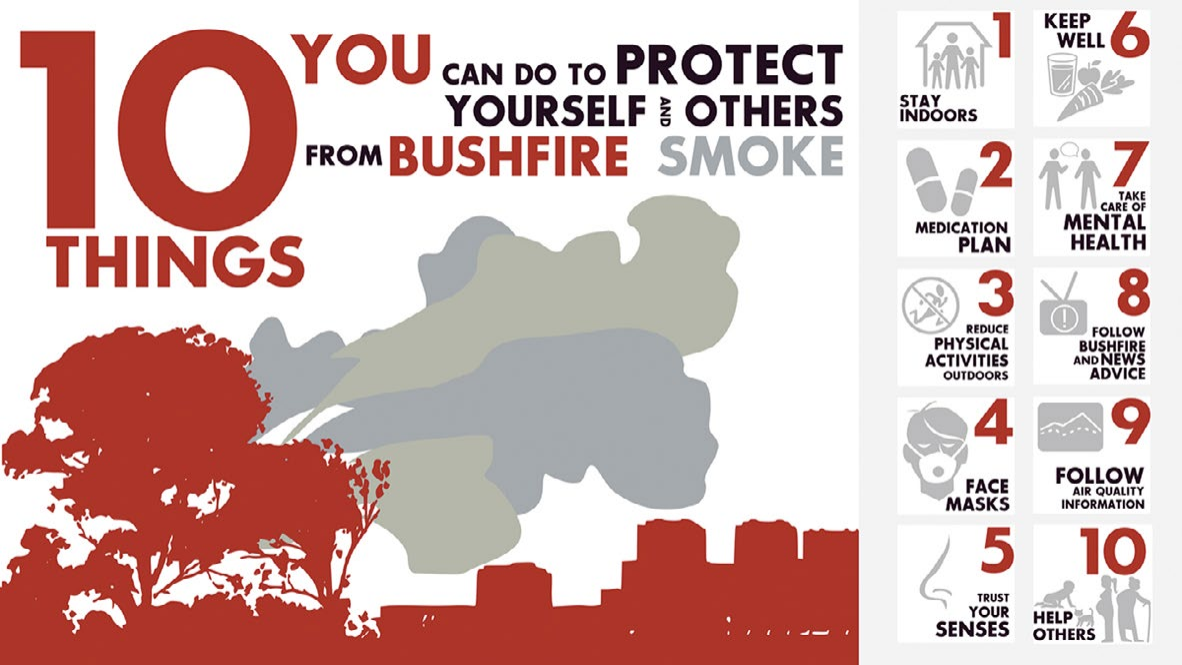

Environmental health literacy and a better understanding of the causes and effects of bushfires, and of the health consequences of air pollution more broadly, are important. There may be a misconception that smoke from burning wood or other organic fuels is “natural”, hence not harmful to health. There is no consistent scientific evidence supporting this belief. Toxicological studies have consistently demonstrated that particles derived from biomass burning can activate inflammatory, oxidative and genotoxic responses, similar to road traffic particles.^23^ A recent systematic review of epidemiological studies has shown higher asthma‐related effects for PM_2.5_ from landscape fire smoke compared with other sources.^11^

Comparison of pollutant concentrations with regulatory standards (eg, 24‐hour PM_2.5_ average of 25 μg/m^3^) highlights the scale of the problem and drives institutional action. However, local air quality can change very rapidly. At a personal level, real time hourly PM_2.5_ data and smoke forecasts are more helpful for planning daily activities to reduce exposure to air pollution. The AirRater smartphone app (https://airrater.org/) shares location‐specific hourly PM_2.5_ measurements from all jurisdictions. However, many locations affected by bushfire smoke do not have air quality monitoring stations. This highlights the need for increased air quality monitoring capabilities at state and territory level, including fixed monitoring sites, portable equipment and low cost sensors that can be rapidly deployed in a bushfire emergency.

It should be emphasised that there is no safe level of exposure to PM_2.5_ and any reduction in exposure reduces the risk of mortality and morbidity. Health professionals often compare outdoor air pollution with cigarette smoke, as both contain mixtures of toxic chemicals and have the same route of exposure (ie, inhalation) and common health outcomes (eg, lung cancer and other respiratory illnesses, heart disease, mortality risk). Although equivalence of bushfire smoke exposure with smoking a specific number of cigarettes is debatable,^24^ the broader comparison helps raise awareness of the long term health risks associated with outdoor air pollution, and reinforces preventive measures.

## Conclusions, recommendations and evidence needs

The unprecedented bushfire smoke levels in eastern Australia have raised concerns about short and long term health consequences in the affected populations. They have also tested the existing health protection advice, which mainly focuses on shorter and more localised smoke episodes, and methods for communicating air quality information. Exposed populations increasingly seek advice on interventions (eg, facemasks, air cleaners, daily activities) that can help people self‐manage health risks from bushfire smoke. It is important that health professionals and patients, as well as healthy individuals and those at higher risk (eg, pregnant women and older people), develop a good understanding of the available health protection measures and their effectiveness and potential trade‐offs (Box 3).^25^, ^26^

Box 3 **Benefits and drawbacks of personal risk reduction measures during bushfire smoke events**

**Table:** 

Risk reduction measure	Benefits	Drawbacks
Staying indoors (at home, workplace or school)^17^	Effective in reducing personal exposure to PM_2.5_ in relative well sealed rooms with: air conditioning (on recirculating mode) air filtration (with HEPA filters) no indoor pollution sources (eg, cigarette smoking)^17^	Building overheating and low air exchange rates resulting in high indoor temperatures and carbon dioxide levels* Significant upfront cost for installation of air conditioning/filtration systems^5^ Ineffective over longer periods of time (ie, several days) without additional air filtration^5^
Reducing strenuous physical exercise outdoors^17^	Effective in reducing personal exposure to bushfire smoke* Limiting exertion in children may be especially important for reducing their exposure to particles^17^	Could be detrimental to cardiovascular and mental health if air pollution persists over longer periods, unless other opportunities for exercising are provided (eg, indoor sports centres)^17^
Using a clean air facility or public building with good indoor air quality (eg, air conditioned shopping centre, public library, community centre, sports centre)*	Effective in reducing exposure to outdoor air pollution over short periods (ie, hours)*	Impractical over longer periods of time (ie, several hours)* At‐risk individuals may need onsite medical assistance or ambulance transport* Large numbers of facilities will be required in cities* Facilities may need retrofits for airtightness or installation of HEPA filters for air intake*
Portable air cleaners (air purifiers)^17^	Effective in reducing indoor air pollution levels if fitted with HEPA filters^5^, ^6^, ^7^, ^8^, ^9^, ^10^, ^11^, ^12^, ^13^, ^14^, ^15^, ^16^, ^17^ Highly effective in well sealed rooms of certain size as recommended by manufacturer^5^, ^6^, ^7^, ^8^, ^9^, ^10^, ^11^, ^12^, ^13^, ^14^, ^15^, ^16^, ^17^	Less effective in less airtight houses, which are common in Australia* PM_2.5_ removal rate dependent on flow rate of air cleaner^17^ Significant upfront purchase cost Availability may be limited in areas heavily affected by bushfire smoke*
Face masks, including professional masks and surgical masks^20^, ^21^, ^22^	Well‐fitted professional (eg, P2/N95) masks offer effective protection from PM_2.5_ exposure^20^ Professional masks are generally suitable for outdoor workers* Exhalation valves can reduce build‐up of humidity and carbon dioxide within masks*	Difficult to achieve good facial fit with professional masks (eg, due to small face, facial hair, etc)^20^ No professional masks are made for children Surgical masks offer only moderate protections^20^ Improvised cloth masks, bandanas or t‐shirts offer no protection^20^ Face masks may give false sense of security^22^ Uncomfortable to wear over longer periods*
Antioxidant supplements, fish oils (omega‐3 fatty acids), and other dietary advice^18^, ^19^	Suggestive evidence that carotenoids and vitamins D and E may protect against pollution damage which can trigger asthma, chronic obstructive pulmonary disease and lung cancer initiation^18^ Vitamin C, curcumin, choline and omega‐3 fatty acids may also have a protective role^18^, ^19^ A healthy diet, rich in fruits and vegetables, is generally beneficial (however, there is no direct evidence of protective effect of diet against air pollution)^18^	Dietary supplements can provide long term and potentially short term health benefits but may be costly* Supplements should not be used as substitute for a healthy and balanced diet* More research is needed to prove effectiveness of supplementation in reducing health risks from air pollution exposure*
Asthma medication, aspirin, statins, other medications^17^	Asthma preventive medication can attenuate exacerbations of the condition* There is very little evidence that aspirin, statins or any other medication have direct protective effects against air pollution^17^	Advance notice of smoke events is required to enable asthma preventive medication to be used*
Smoke forecasts, near real time air quality data (PM_2.5_), air pollution and health alerts^5^	Mostly free to use and can enable individuals to develop personal smoke exposure reduction plans* Localised hourly air quality information more useful than 24‐hour rolling averages or spatially averaged data*	Plethora of air quality websites, apps and indicators, which are not always well validated* Information in electronic media may not reach some sensitive groups (eg, older people)*
Temporary relocation^16^	Can provide health protection to at‐risk groups, such as pregnant women, or people with serious lung or heart disease, affected by localised but persistent smoke episodes*	Impractical when large population centres are affected* Difficult and expensive to relocate many people* Socio‐economically deprived individuals, older people and those who are very ill have lower ability to relocate safely^16^ Cognitive impairment and restricted mobility could compound the stress of relocation^16^

HEPA = high efficiency particulate air. PM_2.5_ = atmospheric particulate matter with a diameter < 2.5 μm. * Based on the authors’ expert opinion.

Public access to local, user‐friendly air quality information and reliable smoke forecasts is essential for managing personal exposure as well as clinical deterioration in sensitive individuals. We strongly recommend that all Australian jurisdictions present actual hourly PM_2.5_ data rather than an index. Real time, hourly averaged PM_2.5_ concentrations are the most appropriate metric to guide personal behaviour that minimises exposure to bushfire smoke. Health messages need to be evidence‐informed and specific for at‐risk groups and the general public. More government investment is needed in air quality monitoring, forecasting and research on public health messaging, and exposure reduction measures to protect Australians from bushfire smoke.

Consistency of air quality information and related public health advice across jurisdictions is essential. It is time for an independent national expert committee on air pollution and health protection to be established to support environmental health decision making in Australia. This new expert committee should have a clear mandate and resources to develop evidence‐based, accurate, practical and consistent advice on health protection against bushfire smoke, and air pollution more broadly, across jurisdictions.

Managing the health impacts of fire smoke should be integral to landscape fire planning and bushfire emergency response. Close collaboration between health, education, environmental, fire management and emergency response agencies is essential for achieving the best overall outcomes for population health and wellbeing. Further research is needed into the medium and longer term impacts of bushfire smoke, as well as the effectiveness and health equity implications of related health protection advice. Working towards ambitious climate change mitigation targets is an essential long term strategy for managing the underlying causes of the increasing bushfire risk in Australia and overseas.

## Competing interests

Sotiris Vardoulakis has received funding support from the UK National Institute for Health Research, Medical Research Council, Natural Environment Research Council, Public Health England, EU Horizon 2020, and Dyson Ltd. Geoffrey Morgan and Ivan Hanigan receive funding support from the Australian National Health and Medical Research Council.

## Provenance

Not commissioned; externally peer reviewed.
